# Increasing atmospheric evaporative demand across the Tibetan plateau and implications for surface water resources

**DOI:** 10.1016/j.isci.2024.111623

**Published:** 2024-12-18

**Authors:** Shiqin Xu, Dennis P. Lettenmaier, Tim R. McVicar, Pierre Gentine, Hylke E. Beck, Joshua B. Fisher, Zhongbo Yu, Ningpeng Dong, Akash Koppa, Matthew F. McCabe

**Affiliations:** 1 Hydrology, Agriculture and Land Observation (HALO) Laboratory, Division of Biological and Environmental Science and Engineering, King Abdullah University of Science and Technology, Thuwal, Saudi Arabia; 2 Climate and Livability Initiative, Division of Biological and Environmental Science and Engineering, King Abdullah University of Science and Technology, Thuwal, Saudi Arabia; 3 Department of Geography, University of California, Los Angeles, Los Angeles, CA, USA; 4 CSIRO Environment, Canberra, ACT, Australia; 5 Australian Research Council Centre of Excellence for Climate Extremes, Canberra, ACT, Australia; 6 Department of Earth and Environmental Engineering, Columbia University, New York, NY, USA; 7 Climate and Livability Initiative, Physical Sciences and Engineering, King Abdullah University of Science and Technology, Thuwal, Saudi Arabia; 8 Schmid College of Science and Technology, Chapman University, Orange, CA, USA; 9 National Key Laboratory of Water Disaster Prevention, Hohai University, Nanjing, P.R. China; 10 College of Hydrology and Water Resources, Hohai University, Nanjing, P.R. China; 11 State Key Laboratory of Simulation and Regulation of Water Cycle in River Basin, China Institute of Water Resources and Hydropower Research, Beijing, P.R. China; 12 Laboratory of Catchment Hydrology and Geomorphology, School of Architecture, Civil and Environmental Engineering, EPFL Valais Wallis, Sion, Switzerland

**Keywords:** Earth sciences, Atmospheric science, Earth-surface processes, Hydrology

## Abstract

The Tibetan Plateau, known as the “Asian water tower,” is a hotspot for complex hydroclimatic changes. We reveal that the previously decreasing atmospheric evaporative demand (*E_o_*) trend at the end of the 20^th^ century has reversed over the last two decades. Although both wind stilling and solar dimming have persisted, their effects on *E*_*o*_ rates have been overshadowed by increasing air temperatures and decreasing relative humidity, leading to a net rise in *E*_*o*_ for 1980−2015. Using the empirical “top-down” Budyko approach, we estimate that across seven sub-catchments draining the Tibetan Plateau, a 10% increase in annual-averaged precipitation, assuming all other factors remain constant, would lead to a 15%–19% increase in streamflow. Conversely, a 10% increase in annual-averaged *E*_*o*_ would decrease streamflow by 5%–9%. Our findings provide a deeper understanding of the accelerating hydroclimatic changes and their impact on surface water resources in the Tibetan Plateau.

## Highlights

• *E*_*o*_ trend considerably increased in 1980–2015 over the TP
• Rising *T*_*a*_ was the major driver of *E*_*o*_ trend
• A 10% increase in annual-averaged *E*_*o*_ would decrease streamflow by 5%–9%

## Graphical abstract

**Figure:**
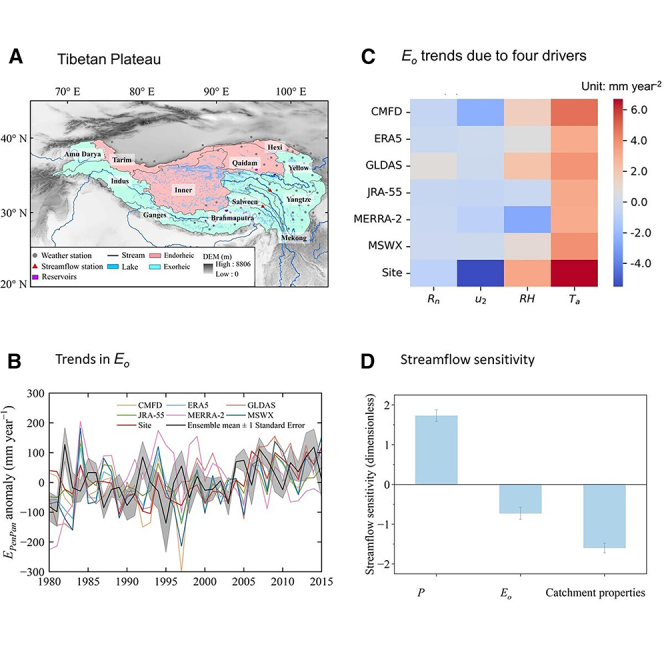

## Introduction

Covering more than 2.5 million km^2^, the Tibetan Plateau (TP) is often referred to as the Asian water tower. It provides freshwater to an estimated two billion people^1^ and delivers a relatively reliable water supply to downstream rivers due to the buffering capacity of its extensive spread of permafrost (∼40% of the TP) and seasonally frozen ground (∼56% of the TP),^2^ snow cover (∼20% of the TP),^3^ glaciers (97,605 km^2^),^4^ and alpine lakes (∼50,000 km^2^ in total).^5^ Climate change is expected to stress the equilibrium of these storage elements^6^^,^^7^^,^^8^ and threatens the downstream hydrology, food security, hydropower, ecosystems, and industries that depend on water resources generated from the TP.^9^

Due to its high elevation (an average elevation of 4,000 m above sea level), the TP has a strong mechanical influence on atmospheric circulation (i.e., westerlies and Asian monsoon system).^10^ It also serves as a strong heat source in summer, as the TP receives more solar radiation than other adjacent non-elevated surfaces.^11^ Any changes in its climate are therefore likely to have an impact on much larger regions within the northern hemisphere, extending well beyond just the neighboring areas.^12^^,^^13^^,^^14^ From 2000 to 2015, the TP experienced considerable climatic change, including, but not limited to, general rapid warming (twice the global average rate) with elevation dependence,^15^^,^^16^ rising vapor pressure deficit,^17^^,^^18^ solar dimming,^19^ and wind stilling.^20^

Most hydrologic studies of the major headwaters of the TP have focused on the impacts of climate change on the supply side of the water balance. Observed changes include a slight overall increase in annual precipitation and contrasting spatial changes,^19^^,^^21^ overall decrease in snow water equivalent,^22^^,^^23^ accelerating glacier melting,^24^^,^^25^^,^^26^ expanding lake area, and increasing lake water storage in the central TP,^5^^,^^27^ along with decreased terrestrial water storage.^28^

Atmospheric evaporative demand (*E*_*o*_), the conceptual upper limit of water flux leaving the land surface (including from vegetation) going into the atmosphere if water were not limiting, plays a fundamental role in climate-induced changes of the hydrological cycle. In addition, *E*_*o*_ is a useful concept both for ecological and agricultural irrigation. At the end of the 20^th^ century, decreasing rates of *E*_*o*_ were widely observed in the TP and its subdomains, with wind stilling and solar dimming identified as the dominant factors causing this *E*_*o*_ reduction (see Table S1 for a detailed literature review). Given that substantial climatic change calls into question the stationarity of the hydrological cycle,^29^ variations in *E*_*o*_ make it crucial to understand the demand component of the terrestrial water budget.^30^^,^^31^

A fundamental understanding of the variation of *E*_*o*_ in space and time and its impacts on surface water resources across the TP is critical because of its implications for both scientific (i.e., monitoring and predicting hydrological changes) and practical (i.e., adapting to climate extremes, irrigation, and water resource management) applications. Considering that the warming rate in the TP tripled from 0.16°C decade^−1^ during 1955–1996^32^ to 0.42°C decade^−1^ during 1980–2015^33^ and the wind stilling has recovered in some locations,^34^ it is critical to close the knowledge gap on the changes in *E*_*o*_ and its hydrological implication in recent decades.

Given both the rapid changes and balances—and counterbalances—among opposing drivers in the Tibetan Plateau, we seek to address the following questions: (1) how has *E*_*o*_ changed in space and time over 1980–2015? (2) which driver (net radiation [*R*_*n*_], wind speed [*u*_*2*_], relative humidity [*RH*], and air temperature [*T*_*a*_]) dominates the *E*_*o*_ changes? and (3) what do *E*_*o*_ changes imply for potential changes of surface water resources under a changing climate?

## Results and discussion

### Spatial and temporal trends in *E*_*o*_

**Figure 1:**
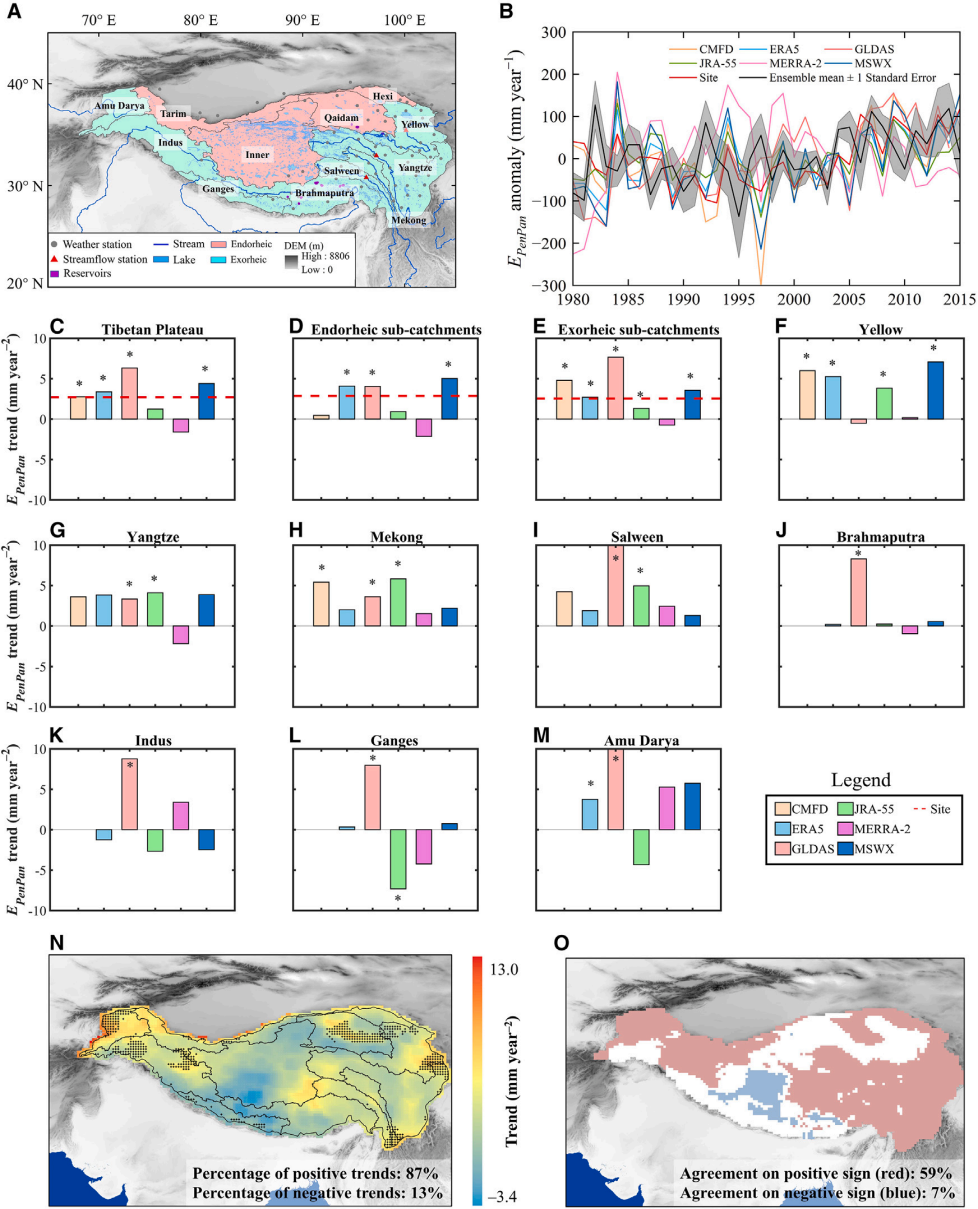
Trends in annual E_*PenPan*_ across the TP for 1980–2015 (A) Sub-catchments within the TP, (B) interannual variability of *E*_*PenPan*_ anomaly, (C–M) *E*_*PenPan*_ trends in the entire TP and sub-catchments forced with both site observations and six gridded datasets; (N) spatial distribution of *E*_*PenPan*_ trends averaged from gridded datasets, and (O) a consistency map where at least four out of the five datasets (i.e., ERA5, GLDAS, JRA-55, MERRA-2, and MSWX) agree on an increase (red) or decrease (blue). Given the limited and uneven distribution of sites, we opted not to include site-specific observations in the analysis at exorheic sub-catchments. Stippling in (N) denotes grids where at least four out of the five corresponding datasets exhibit a significant trend (*p* value <0.05). Site *E*_*PenPan*_ showed significant increasing trends (*p* value <0.05) across the entire TP and the endorheic and exorheic sub-catchments. The code “∗” indicates that trend in *E*_*PenPan*_ from that data was significant (*p* value <0.05). The legend to the right of (M) applies to parts (C)–(M). The regional-averaged ensemble means of annual *E*_*PenPan*_ derived from both site observations and gridded datasets are represented as mean ± standard error.

The time series of pan evaporation, as estimated by the PenPan model^35^ (denoted *E*_*PenPan*_), consistently show an increasing trend across the entire TP for 1980–2015 when using different datasets, with the exception of MERRA-2 (Figures 1A and 1B). Estimates from GLDAS show the greatest rate of increase (+6.3 mm year^−2^), followed by the rate from MSWX (+4.4 mm year^−2^), ERA5 (+3.4 mm year^−2^), CMFD (+2.8 mm year^−2^), weather stations (+2.7 mm year^−2^), and JRA-55 (+1.2 mm year^−2^), respectively (Figure 1C and Table S2). Five datasets (GLDAS, MSWX, ERA5, CMFD, and site observations) with considerable increasing trends are field significant (*p* value <0.05). Time series of the other three *E*_*o*_ metrics (i.e., *E*_*o*_ calculated from Penman^36^ [denoted *E*_*Penman*_], Priestley-Taylor^37^ [denoted *E*_*PT*_], and FAO-56 reference crop^38^ [denoted *ET*_*RC*_] models) also notably increased over the TP at lower rates, in agreement with the *E*_*PenPan*_ results (see Figure S1 and Tables S3–S5). Considering the physiological effects of increasing atmospheric CO_2_ on vegetation,^39^^,^^40^ we further applied the CO_2_-adjusted FAO-56 reference model^41^ (donated as *ET*_*RC,CO2*_) to estimate *E*_*o*_ and the corresponding trends. The result was consistent with the findings presented in Figure 1B, although the count of sub-catchments exhibiting significant trends (*p* value <0.05) slightly decreased (see Figure S1C and Table S6).

Spatially, *E*_*PenPan*_ increased substantially both in the endorheic and exorheic sub-catchments (Figures 1D–1M). Increasing trends in *E*_*PenPan*_ of these datasets showed high consistency in the Yellow, Yangtze, Mekong, Salween, Brahmaputra, and Amu Dayra River sub-catchments. Trends in *E*_*PenPan*_ were positive for 87% of the TP pixels for the ensemble mean of the five datasets with 10% being statistically significant (*p* value <0.05; Figure 1N). Only 13% of the pixels covering the TP had negative *E*_*PenPan*_ trends. Given the spatial variability of *E*_*PenPan*_ trends in the sub-catchments across these datasets (Figures 1C–1M), we further defined a consistency map by denoting the trends in *E*_*PenPan*_ with stippling when at least four out of the five gridded datasets agree on sign. Overall, 59% of the TP exhibited consistent increases and only 7% showed decreases, primarily concentrated in the inner TP and upper Brahmaputra (Figure 1O). The sign agreement of the trends across the TP from the multiple widely used datasets indicates that our detection of changes in *E*_*o*_ are robust.

### Attributing *E*_*o*_ changes

**Figure 2:**
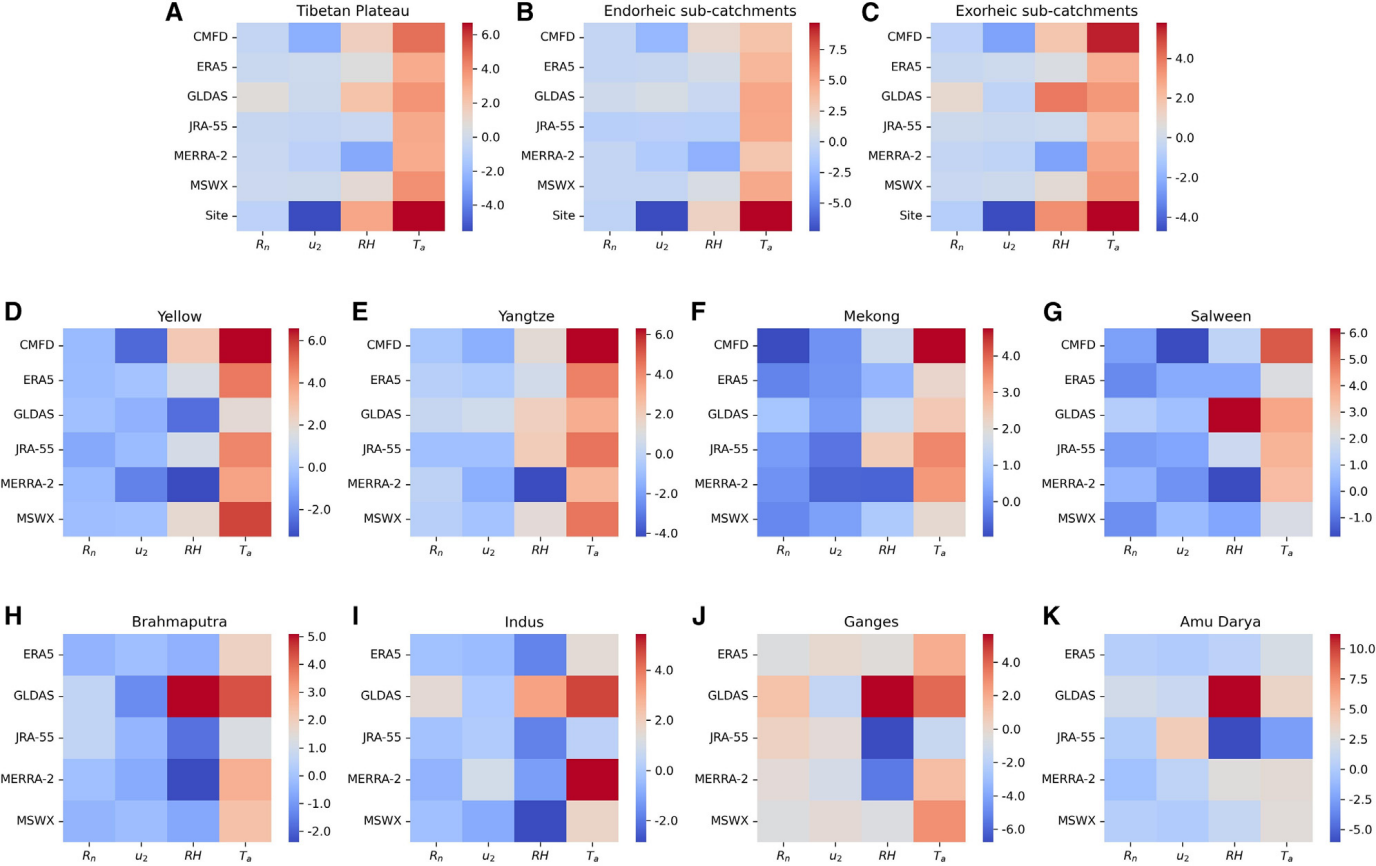
Attribution of changes in *E*_*PenPan*_ for 1980–2015 due to changing *R*_*n*_, *u*_*2*_, *RH*, and *T*_*a*_ over the entire TP and sub-catchments (A) Tibetan Plateau, (B) Endorheic sub-catchments, (C) Exorheic sub-catchments, and (D–K) Yellow, Yangtze, Mekong, Salween, Brahmaputra, Indus, Ganges, and Amu Darya River sub-catchments, respectively. All units are in year^−2^.

We revealed that *E*_*o*_ calculated from different datasets and models considerably increased over the past four decades. The following question arises: which driver dominates the *E*_*o*_ changes in the TP? Figure 2 compares the contributions of the four meteorological drivers (i.e., *R*_*n*_, *u*_*2*_, *RH*, and *T*_*a*_) to changes in *E*_*PenPan*_ across the entire TP and sub-catchments. The site observations show that most of the trends in *E*_*PenPan*_ are attributable to changes in *T*_*a*_ (Figure 2A). The results of attributing the changes in observed *E*_*PenPan*_ to four climate drivers are, in order of absolute magnitude, due to: (1) $\frac{dT_{a}}{dt}$ (+6.7 mm year^−2^), (2) $\frac{du_{2}}{dt}$ (−5.5 mm year^−2^), (3) $\frac{dRH}{dt}$ (+3.2 mm year^−2^), and (4) $\frac{dR_{n}}{dt}$ (−0.8 mm year^−2^). All of the gridded datasets identified rising *T*_*a*_ as the major driver of *E*_*PenPan*_ trends over 1980–2015 for the entire TP (+4.8 of CMFD; +3.9 of MSWX; +3.8 of GLDAS; +3.1 of ERA5, +3.1 of JRA-55, and +3.1 of MERRA-2; all units are in mm year^−2^) (Figure 2A). Rising *T*_*a*_ was also identified as the dominant driver of *E*_*PenPan*_ changes in most sub-catchments (Figures 2B–2K). However, exceptions were noted for the Brahmaputra, Indus, and Ganges sub-catchments, where gridded datasets identified different dominant meteorological drivers (Figures 2H–2J).

The recent dominance of *T*_*a*_ in regulating *E*_*o*_ changes is consistent with analysis reported from other parts of the globe. For example, revisiting *E*_*PenPan*_ trends in Australia for 1975–2016 (42 years) showed that many previously reported decreasing tendencies in *E*_*PenPan*_ for 1975–2004^42^ plateaued or reversed.^43^ Increasing vapor pressure deficit due to rising *T*_*a*_ exerted a greater influence than *u_2_* changes,^43^ which is consistent with our TP findings. Furthermore, gridded datasets underestimate the contribution of changes in *u*_*2*_ to *E*_*PenPan*_ trends in comparison with site estimates, except for CMFD that assimilated more site observations (Figures 2A–2C). This is probably because terrestrial wind stilling is either not reproduced well or has been underestimated in most global reanalysis products.^44^^,^^45^

### Implications of *E*_*o*_ trends for surface water resources

What do accelerated *E*_*o*_ changes imply for potential changes in surface water resources of the TP? To answer this question, we use the “top-down” Budyko modeling approach^46^ to investigate the sensitivity of streamflow (*Q*) to changes in precipitation (*P*), *E*_*o*_, and catchment properties (*n*). We focus on *Q* because it is a key regulator of water resources for society and a critical hydrological variable for diagnosing climatic change.

Previous studies reported an overall wetting trend and a north-south dipole pattern in *P* changes.^19^^,^^24^ We revisited the trends in *P* over the entire TP and sub-catchments based on multi-source datasets with extended temporal period (Figure S2). From 1980 to 2015, the entire TP, endorheic and exorheic Yellow, Yangtze, Mekong, and Salween sub-catchments exhibited increasing *P* (see Figures S2A–S2G) due to the strengthening of prevailing mid-latitude westerlies.^47^ In contrast, the Indus and Amu Darya sub-catchments experienced decreasing *P* because the Indian monsoon weakened since the 1980s^48^^,^^49^ (see Figures S2I and S2K). Although most of the gridded datasets showed significant increasing trends (*p* value <0.05) in the entire TP and sub-catchments, observed *P* increased insignificantly (*p* value >0.05) (see Figures S2A–S2C). Moreover, the Brahmaputra and Ganges sub-catchments exhibited divergent *P* trends across these grided datasets (see Figures S2H and S2J). The discrepancy in trend sign between site-specific and gridded datasets, along with variations across different datasets in the southern TP, highlights the ongoing challenges associated with ground *P* estimates in mountainous regions.^50^

**Figure 3:**
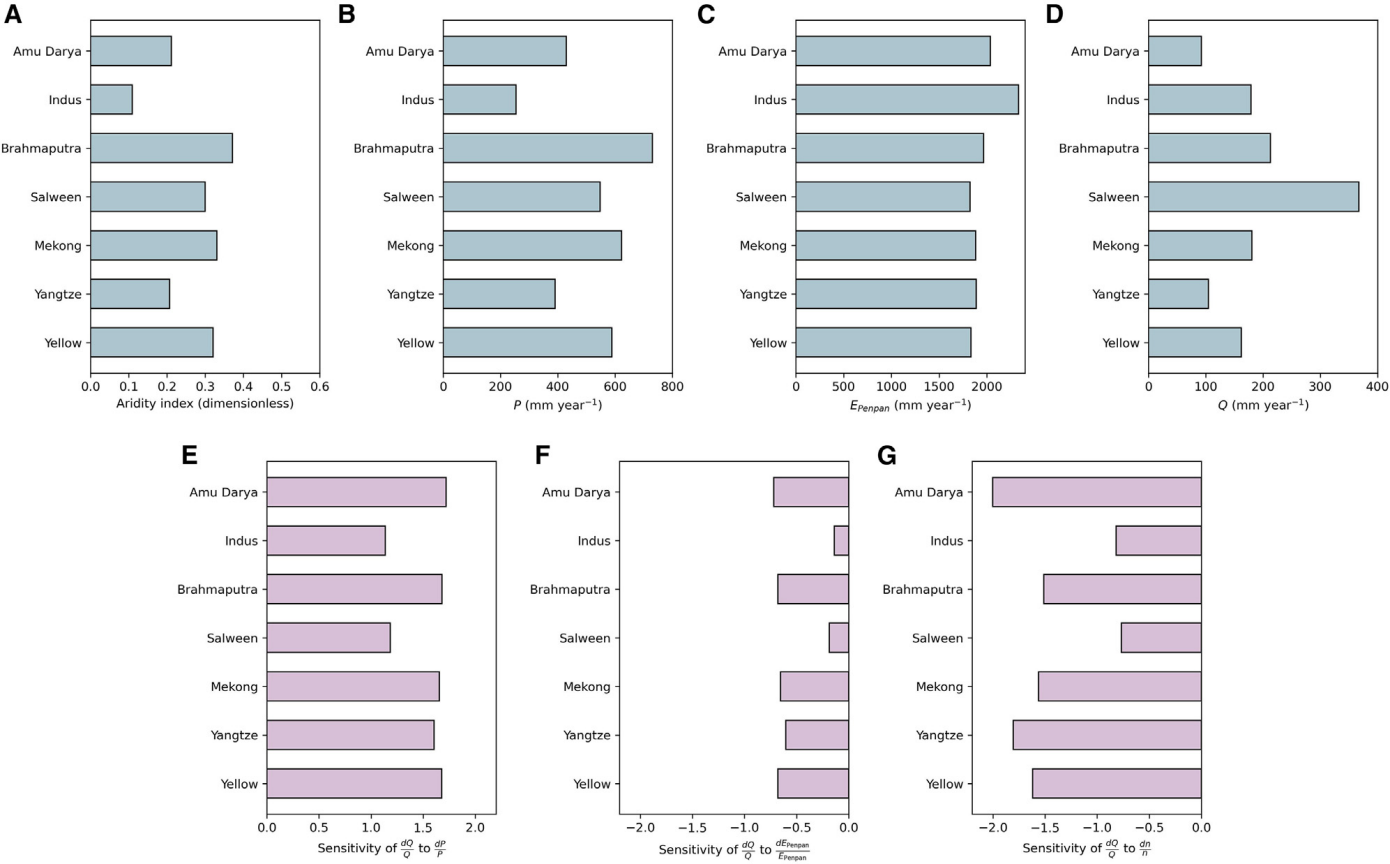
Sensitivity of *Q* to changes in climate (*P*, *E*_*PenPan*_) and catchment properties (*n*) in the seven TP-heading river sub-catchments (A–D) Long-term climatology over 1980–2015 of aridity index ($=\frac{P}{E_{PenPan}}$), *P*, *E*_*PenPan*_, and *Q*. (E–G) Sensitivity of relative changes in *Q* (denoted $\frac{dQ}{Q}$) to the relative change in *P* (denoted $\frac{dP}{P}$), *E*_*PenPan*_ (denoted $\frac{dE_{PenPan}}{dE_{PenPan}}$), and *n* (denoted $\frac{dn}{n}$). Noting that if *P* gridded data had obvious biases (i.e., the value was more than or less than 30% of ensemble mean), then it was rejected for a specific catchment.

Seven exorheic sub-catchments showed considerable difference in climatology in *P*, *E*_*PenPan*_, and *Q*, despite having a typical arid climate (Figures 3A–3D). *Q* is predicted to be more sensitive to a change in *P* than to comparable changes in *E*_*PenPan*_ in those sub-catchments (Figures 3E–3G). This result is typical of arid environments where *Q* is largely controlled by water availability (i.e., *P*) in the long term. Our theoretical result for those rivers predicts that a 10% increase in *P*, with all else constant, would lead to a 10%–17% increase in *Q*, whereas a 10% increase in *E*_*PenPan*_ would decrease *Q* by 2%–7%. The sensitivity of *Q* to changes in catchment properties, *n*, has a comparable range of values to changes in *P*, and a 10% change in *n* leads to an 8%–20% decrease in *Q*. Integrating regionally over the seven sub-catchments for which *Q* were available, a 10% increase in *P* would lead to a 15%–19% increase in *Q*, a 10% increase in *E*_*o*_ would decrease *Q* by 5%–9%, and a 10% increase in *n* would decrease *Q* by 14%–17% when different *E*_*o*_ metrics are being used (see Table S7).

The ‘Budyko’ approach has experienced a renaissance since the early 2000s, with many assessments of the impacts of climate change on water resources based on his framework.^51^^,^^52^^,^^53^ However, few studies^54^ have assessed the impacts of selecting different *E*_*o*_ metrics on the sensitivity quantification. We found that although the rank of sensitivity of *Q* to *P*, *E*_*o*_, and *n* remain the same when different *E*_*o*_ models were used, the magnitude of the sensitivity to three factors varied considerably. For example, applying the Penman^36^ or a simplified radiation-driven model^55^ produced similar results to those from the PenPan model.^35^ However, when Priestley-Taylor,^37^ FAO-56 reference crop,^38^ or CO_2_-adjusted FAO-56 reference crop^41^ model was used, the sensitivity of *Q* to changes in *E*_*o*_ increased (see Table S7). The results indicate that the parameterization of *E*_*o*_ can have considerable effects on hydrological modeling.

A continuous rise in *T*_*a*_ is expected with a warming climate across the TP.^47^ Projected changes in *P* are more uncertain with larger inter-model spread and higher spatial variability.^9^^,^^47^ Climate change could therefore compound the effects of water stress on surface water resources through combined accelerated evaporation demand and decreased water supply and streamflow, which is likely to exacerbate ecosystem and agricultural stress.

### Limitations of the study

Individual gridded datasets exhibit inconsistency in their *E*_*o*_ changes across certain regions of TP. For example, trends in *E*_*PenPan*_ across datasets were inconsistent in the Indus and Ganges sub-catchments. Spatially, 34% of the TP exhibited divergent trends among gridded datasets, primarily concentrated in the inner TP and Qaidam sub-catchment. Moreover, site-specific and gridded datasets showed discrepancies in the sign of the *P* trend. These inconsistencies highlight the ongoing challenges in accurately estimating spatial variations of trends in atmospheric covariates across the TP.

## Resource availability

### Lead contact

Further information and requests for resources and reagents should be directed to and will be fulfilled by the lead contact, Shiqin Xu (shiqin.xu@hotmail.com).

### Materials availability

This study did not generate new unique materials.

### Data and code availability

• Data: this paper analyzes existing, publicly available data. These accession numbers for the datasets are listed in the key resources table.
• Code: this paper does not report original code.
• Any additional information required to reanalyze the data reported in this paper is available from the lead contact upon request.

## Acknowledgments

S.X. acknowledges support from the 10.13039/100014717National Natural Science Foundation of China (Grant No. 42101045). M.F.M. acknowledges support from the 10.13039/501100004052King Abdullah University of Science and Technology. Two anonymous reviewers provided useful comments and insightful suggestions to this manuscript. We thank the many investigators for allowing us to use their data.

## Author contributions

S.X., D.P.L., and T.R.M. conceived the study with inputs from all co-authors. S. X. and N. D. digitized hydrological yearbooks and collected streamflow observations. S. X. performed data analysis, wrote the first draft of the manuscript, and coordinated the manuscript preparation. All co-authors reviewed and edited the paper.

## Declaration of interests

The authors declare no competing interests.

## STAR★Methods

### Key resources table

**Table:** 

REAGENT or RESOURCE	SOURCE	IDENTIFIER
**Deposited data**		

Weather station observation	China Meteorological Administration	http://data.cma.cn/
Meteorological product	CMFD	https://data.tpdc.ac.cn/zh-hans/data/8028b944-daaa-4511-8769-965612652c49/
Meteorological product	GLDAS	https://disc.gsfc.nasa.gov/datasets/GLDAS_CLSM025_D_2.0/summary?keywords=GLDAS/
Reanalysis	ECMWF ERA5	https://cds.climate.copernicus.eu/
Reanalysis	JRA-55	https://rda.ucar.edu/datasets/ds628.1/
Reanalysis	MERRA-2	https://disc.gsfc.nasa.gov/datasets?project=MERRA-2/
Meteorological product	MSWX	http://www.gloh2o.org/mswx/
Meteorological product	MSWEP V2.0	https://www.gloh2o.org/mswep/
Meteorological product	CHIRPS V2.0	https://data.chc.ucsb.edu/products/CHIRPS-2.0/
Global river discharge reanalysis	GloFAS-ERA5	https://data.jrc.ec.europa.eu/collection/id-00288/
Global digital elevation model	GTOP30	https://www.usgs.gov/centers/eros/science/usgs-eros-archive-digital-elevation-global-30-arc-second-elevation-gtopo30/
Tibet Plateau boundary	TPDC	https://data.tpdc.ac.cn/home/

**Software and algorithms**		

MATLAB	MathWorks	https://www.mathworks.com/products/matlab.html
Microsoft EXCEL	Microsoft	https://www.microsoft.com/ja-jp/microsoft-365/excel

### Method details

#### Study area

The TP is the world’s largest (∼250 × 10^4^ km^2^) and highest (averaging over 4,000 m a.s.l) Cenozoic plateau.^47^ Extensive snow coverage, permafrost and seasonally frozen ground, glaciers, alpine lakes in the eight major river headwater catchments (the Yellow, Yangtze, Mekong, Salween, Brahmaputra, Ganges, Indus, and Amu Dayra Rivers) supply freshwater to their downstream reaches (see Table S8 for details of these sub-catchments). Hydrological change in the TP is primarily controlled by its large-scale atmospheric circulation, particularly the westerlies and the Asian monsoon system.^56^^,^^57^ The river networks that drain the TP are either endorheic (i.e., those which drain internally) catchments located mostly in the central and northern edge of the TP or exorheic catchments (i.e., those which drain externally, typically rivers which eventually reach the ocean) located mainly in the southern and eastern TP (see Figure 1A).

#### Models

In theory, *E*_*o*_ represents a relatively straightforward concept, but there are more than 50 *E*_*o*_ models.^58^^,^^59^ Here we applied four commonly used approaches: (i) Penman^36^ (denoted *E*_*Penman*_); (ii) Priestley-Taylor^37^ (denoted *E*_*PT*_); (iii) FAO-56 reference crop^38^ (denoted *ET*_*RC*_); and (iv) PenPan^35^ (denoted *E*_*PenPan*_) models. The Penman model is a fully physically based form of *E*_*o*_; the PT model is a bi-variate radiative model of *E*_*o*_; FAO-56 quantifies a reference crop water use for a prescribed set of reference crop conditions; and PenPan is a model of pan evaporation. Additionally, we used the CO_2_ adjusted FAO-56 reference model^41^ (donated as *ET*_*RC,CO2*_) that accounts for the CO_2_ effect on surface resistance to estimate *E*_*o*_, and a radiation-driven model.^55^

##### Penman model

The Penman equation^36^ for estimating open-water evaporation is defined as follows:

(Equation 1) $$E_{Penman}=\frac{\Delta }{\Delta +\gamma }\frac{R_{n}}{\lambda }+\frac{\gamma }{\Delta +\gamma }E_{a}$$

where *E*_*Penman*_ is the daily open-water evaporation (day mm^−1^), *R*_*n*_ is net daily radiation at the water surface (MJ m^−2^ day^−1^), *E*_*a*_ (mm day^−1^) is a function of the average daily wind speed (m s^−1^), saturation vapor pressure (kPa) and average vapor pressure (kPa), Δ is the slope of the vapor pressure curve (kPa °C^−1^) at air temperature, γ is the psychrometric constant (kPa °C^−1^), and λ is the latent heat of vaporization (MJ kg^−1^).

To estimate *E*_*a*_ in Equation 1, one should use:

(Equation 2) $$E_{a}=f\left(u\right)\left(e_{s}-e_{a}\right)$$

where f(u) is the wind function and $\left(e_{s}-e_{a}\right)$ is the vapor pressure deficit (kPa). We adopted Penman’s equation^36^ as standard:

(Equation 3) $$f\left(u\right)=2.626+1.38u_{2}$$

where *u*_*2*_ is the average daily wind speed (m s^−1^) at the height of 2 m. The following equation was used to estimate *u*_*2*_.

(Equation 4) $$u_{2}=u_{z}\frac{\log\;\left(\frac{2}{z_{0}}\right)}{\log\;\left(\frac{z}{z_{0}}\right)}$$

where *u*_*2*_ and *u*_*z*_ are the wind speed (m s^−1^) at the height of 2 m and *z* m, respectively, and *z*_*0*_ is the roughness height ($z_{0}=0.001$).

##### Priestley-Taylor model

The Priestley-Taylor equation^37^ allows *E*_*o*_ to be computed in terms of energy fluxes with the aerodynamic component of *E*_*o*_ assumed to be 0.26 of the radiative component (as the surface is assumed to be extensive and well-watered, so there is assumed to be no advection) and it is calculated as follows:

(Equation 5) $$E_{PT}=\alpha_{PT}\left[\frac{\Delta }{\Delta +\gamma }\frac{R_{n}-G}{\lambda }\right]$$

where *E*_*PT*_ is the Priestley-Taylor potential evaporation (mm day^−1^), *R*_*n*_ is the net daily radiation at the evaporating surface (MJ m^−2^ day^−1^), *G* is the soil flux into the ground (MJ m^−2^ day^−1^), Δ is the slope of the vapor pressure curve (kPa°C^−1^) at air temperature, *γ* is the psychrometric constant (kPa °C^−1^), and *λ* is the latent heat of vaporization (MJ kg^−1^). $\alpha_{PT}$ is the Priestley-Taylor constant and the recommended $\alpha_{PT}=1.26$ was adopted here.

##### FAO-56 reference model

Adopting the characteristics of a hypothetical reference crop (height = 0.12 m, surface resistance = 70 s m^−1^, and *α* = 0.23), the Penman-Monteith equation becomes Equation 6, which is known as the FAO-56 (Food and Agriculture Organization) reference crop evapotranspiration model,^38^ and is defined as follows:

(Equation 6) $${ET}_{RC}=\frac{0.408\Delta \left(R_{n}-G\right)+\gamma \frac{900}{T_{a}+273}u_{2}\left(e_{s}-e_{a}\right)}{\Delta +\gamma \left(1+0.34u_{2}\right)}$$

where *ET*_*RC*_ is the daily reference crop evapotranspiration (mm day^−1^), *R*_*n*_ is the net daily radiation at the land surface (MJ m^−2^ day^−1^), *G* is the soil flux into the ground (MJ m^−2^ day^−1^), *γ* is the psychrometric constant (kPa °C^−1^), *T*_*a*_ is the mean daily air temperature (°C) at the height of 2 m, *u*_*2*_ is the average daily wind speed (m s^−1^) at the height of 2 m, and $\left(e_{s}-e_{a}\right)$ is the vapor pressure deficit (kPa).

Wind speed at 2-m height can be estimated as follows^38^:

(Equation 7) $$u_{2}=u_{z}\frac{4.87}{\ln\;\left(67.8z-5.42\right)}$$

where *u*_*z*_ (m s^−1^) is the wind speed at the height of *z* m, and *z* is the height of measurement above ground surface (m).

##### CO_2_ adjusted FAO-56 reference model

A CO_2_ adjusted FAO-56 reference crop evapotranspiration model that accounts for the CO_2_ effect on surface resistance^41^ is as follows:

(Equation 8) $${ET}_{RC,CO_{2}}=\frac{0.408\Delta \left(R_{n}-G\right)+\gamma \frac{900}{T_{a}+273}u_{2}\left(e_{s}-e_{a}\right)}{\Delta +\gamma \left\{1+u_{2}\left[0.34+2.4\times 10^{-4}\left[CO_{2}\right]-300)\right]\right\}}$$

where the term 2.4 × 10^−4^ ([CO_2_] – 300) accounts for changing CO_2_ on surface resistance.

In the equation, *R*_*n*_ is the net daily radiation at the land surface (MJ m^−2^ day^−1^), *G* is the soil flux into the ground (MJ m^−2^ day^−1^), *γ* is the psychrometric constant (kPa °C^−1^), *T*_*a*_ is the mean daily air temperature (°C) at the height of 2 m, *u*_*2*_ is the average daily wind speed (m s^−1^) at the height of 2 m, and $\left(e_{s}-e_{a}\right)$ is the vapor pressure deficit (kPa).

##### PenPan model

The PenPan model^35^ was developed to estimate evaporation from the Class A pan.

(Equation 9) $$E_{PenPan}=E_{PenPan,R}+E_{PenPan,A}=\frac{\Delta }{\Delta +a_{p}\gamma }\frac{R_{n}}{\lambda }+\frac{a_{p}\gamma }{\Delta +a_{p}\gamma }f_{Pan}\left(u\right)\left[e_{s}\left(1-\frac{RH}{100}\right)\right]$$

where *E*_*PenPan*_ is the estimated pan evaporation (mm day^−1^), *E*_*PenPan,R*_ is the radiative component, *E*_*PenPan,A*_ is the aerodynamic component, *R*_*n*_ is the net daily radiation at the pan (MJ m^−2^ day^−1^), *Δ* is the slope of the vapor pressure curve (kPa °C^−1^) at air temperature (°C^−1^), *γ* is psychrometric constant (kPa °C^−1^), and λ is the latent heat of vaporization (MJ kg^−1^), *α*_*p*_ is a constant adopted as 5 for the China D20 pan,^60^ $e_{s}$ is the saturated vapor pressure (kPa), *RH* is the relative humidity (%), and $f_{Pan\left(u\right)}$ (adjusted for the Chinese D20 pan) is defined as^60^:

(Equation 10) $$f_{Pan}\left(u\right)=5.4\times \left(1+0.73\right)u_{2}$$

where *u*_*2*_ is the average daily wind speed at 2m height (m s^−1^).

*R*_*n*_ at the pan (MJ m^−2^ day^−1^) can be calculated as follows:

(Equation 11) $$R_{n}=\left(1-a_{pan}\right)R_{sp}-R_{nl}$$

where *R*_*sp*_ is the incoming shortwave radiation received by the pan, which is greater than *R*_*s*_ because of additional interception by the walls of the pan.^35^ *R*_*nl*_ is the outgoing net longwave radiation (MJ m^−2^ day^−1^) and $\alpha_{pan}$ is the pan albedo ($\alpha_{pan}=0.14$).

*R*_*sp*_ can be estimated as follows

(Equation 12) $$R_{sp}=\left[P_{rad}f_{dir}+2\left(1-f_{dir}\right)+2\alpha \right]R_{s}$$

where *P*_*rad*_ is a pan radiation factor, *f*_*dir*_ is the fraction of *R*_*s*_ that is direct, and α is the assumed ground surface albedo (α=0.23).

$f_{dir}$^61^ and *P*_*rad*_^60^ are defined as

(Equation 13) $$f_{dir}=-0.11+1.31\frac{R_{s}}{R_{a}}$$

(Equation 14) $$P_{rad}=1.70+3\times 0.0004\times {lat}^{2}$$

where *R*_*a*_ is the extraterrestrial radiation (MJ m^−2^ day^−1^), and lat is the absolute value of latitude in degrees.

##### Radiation-driven model

A radiation-driven model^55^ is as follows:

(Equation 15) $$E_{R_{n}}=0.8\left(R_{n}-G\right)$$

where *R*_*n*_ is the net daily radiation at the evaporating surface (converted from unit MJ m^−2^ year^−1^ to mm year^−1^).

We assessed the performance of PenPan model using pan evaporation observations from weather stations (see Figure S3 for the assessment) and presented the results in the main text.

#### Datasets

To provide a comparative and robust detection and attribution of trends in *E*_*o*_ across the TP, we used seven datasets: (i) observations from data of 79 filtered CMA (China Meteorological Administration) weather stations which are mostly located in the eastern TP (see Figure 1A); (ii) China Meteorological Forcing Dataset (CMFD)^62^; (iii) forcing fields of a Global Land Data Assimilation System (GLDAS)^63^; (iv) ECMWF ERA5^64^; (v) the Japanese 55-year Reanalysis (JRA-55)^65^; (vi) Modern-Era Retrospective Analysis for Research and Applications, version 2 (MERRA-2)^66^; and (vii) the new-released Multi-Source Weather (MSWX) meteorological data,^67^ which was derived by bias correcting the ERA5 reanalysis using high-resolution reference climatologies. We included two additional precipitation products (i.e., MSWEP V2.0^68^ and CHIRPS V2.0^69^) for more accurate estimation on long-term precipitation climatology and trend detection. A summary of these datasets provided in Table S9. Bilinear interpolation was consistently applied to resample gridded datasets. After the homogenization, the gridded datasets share a common spatial-temporal grid of 0.25° in space.

Monthly observed streamflow (*Q*) at the watershed outlet of the Yellow, Yangtze, and Salween River catchments were provided by the Ministry of Water Resources of China. For the Mekong, Brahmaputra, Indus, and Amu Dayra River catchments, where observed streamflow is difficult to access, daily river streamflow from GloFAS-ERA5^70^ were used. An assessment of the GloFAS-ERA5 streamflow data was provided in Figure S4.

#### Trend detection

We used the Mann-Kendall (MK hereafter) statistical test^71^^,^^72^ to detect inter-annual changes of the four *E*_*o*_ metrics. We determined trend magnitudes using the Theil-Sen approach (TSA).^73^^,^^74^ Given that trends in hydro-climatological variables were highly spatially coherent^75^ and to account for this spatial correlation, we used Walker’s test^76^ to determine field significance (*p*-value < 0.05) of the trends.

#### Attributing *E*_*o*_ changes

We performed attribution of *E*_*PenPan*_ trends by differentiating the PenPan model^77^ by the relative rates of changes of the four driving meteorological variables (i.e., *R*_*n*_, *u*_*2*_, *RH*, and *T*_*a*_). Changes in *E*_*PenPan*_ were attributed to the changes in the radiative ($\frac{dE_{PenPan,R}}{dt}$) and aerodynamic ($\frac{dE_{PenPan,A}}{dt}$) components using the relationship:

(Equation 16) $$\begin{array}{c} \frac{dE_{PenPan}}{dt}=\frac{dE_{PenPan,R}}{dt}+\frac{dE_{PenPan,A}}{dt} \end{array}$$

Dynamics in the radiative component of the evaporative demand are due to changes in the slope of Clausius-Clapeyron *Δ*, which is calculated as a function solely of *T*_*a*_.^38^

(Equation 17) $$\begin{array}{c} \frac{dE_{PenPan,R}}{dt}=\frac{\partial E_{PenPan,R}}{\partial \Delta }\frac{d\Delta }{dT_{a}}\frac{dT_{a}}{dt}+\frac{\partial E_{PenPan,R}}{\partial R_{n}}\frac{dR_{n}}{dt} \end{array}$$

where

(Equation 18) $$\begin{array}{c} \frac{\partial E_{PenPan,R}}{\partial \Delta }\frac{d\Delta }{dT_{a}}\frac{dT_{a}}{dt}=\frac{a_{p}\gamma R_{n}}{{\left(\Delta +a_{p}\gamma \right)}^{2}\lambda }\frac{d\Delta }{dT_{a}}\frac{dT_{a}}{dt} \end{array}$$

and

(Equation 19) $$\begin{array}{c} \frac{\partial E_{PenPan,R}}{\partial R_{n}}\frac{dR_{n}}{dt}=\frac{\Delta }{\left(\Delta +a_{p}\gamma \right)\lambda }\frac{dR_{n}}{dt} \end{array}$$

Given that *e*_*s*_ changes only with *T*_*a*_, changes in the aerodynamic component are:

(Equation 20) $$\begin{array}{c} \frac{dE_{PenPan,A}}{dt}=\frac{\partial E_{PenPan,A}}{\partial \Delta }\frac{d\Delta }{dT_{a}}\frac{dT_{a}}{dt}+\frac{\partial E_{PenPan,A}}{\partial u_{2}}\frac{du_{2}}{dt}+\frac{\partial E_{PenPan,A}}{\partial e_{s}}\frac{de_{s}}{dT_{a}}\frac{dT_{a}}{dt}+\frac{\partial E_{PenPan,A}}{\partial RH}\frac{dRH}{dt} \end{array}$$

with the contribution from *Δ*, *u*_*2*_, *e*_*s*_, and *RH* estimated, respectively, as:

(Equation 21) $$\begin{array}{c} \frac{\partial E_{PenPan,A}}{\partial \Delta }\frac{d\Delta }{dT_{a}}\frac{dT_{a}}{dt}=-\frac{a_{p}\gamma \left[5.4\left(1+0.73u_{2}\right)\right]\left[e_{s}\left(1-\frac{RH}{100}\right)\right]}{{\left(\Delta +a_{p}\gamma \right)}^{2}}\frac{d\Delta }{dT_{a}}\frac{dT_{a}}{dt} \end{array}$$

(Equation 22) $$\begin{array}{c} \frac{\partial E_{PenPan,A}}{\partial u_{2}}\frac{du_{2}}{dt}=\frac{a_{p}\gamma \left[e_{s}\left(1-\frac{RH}{100}\right)\right]0.73}{\left(\Delta +a_{p}\gamma \right)}\frac{du_{2}}{dt} \end{array}$$

(Equation 23) $$\begin{array}{c} \frac{\partial E_{PenPan,A}}{\partial e_{s}}\frac{de_{s}}{dT_{a}}\frac{dT_{a}}{dt}=\frac{a_{p}\gamma \left[5.4\left(1+0.73u_{2}\right)\right]\left(1-\frac{RH}{100}\right)}{\Delta +a_{p}\gamma }\frac{de_{s}}{dt}\frac{dT_{a}}{dt} \end{array}$$

(Equation 24) $$\begin{array}{c} \frac{\partial E_{PenPan,A}}{\partial RH}\frac{dRH}{dt}=-\frac{a_{p}\gamma \left[5.4\left(1+0.73u_{2}\right)\right]\frac{e_{s}}{100}}{\Delta +a_{p}\gamma }\frac{dRH}{dt} \end{array}$$

To bring the attribution back to the four key driving meteorological variables of *E*_*PenPan*_ (i.e., *R*_*n*_, *u*_*2*_, *RH*, and *T*_*a*_), the effect of $\frac{dT_{a}}{dt}$ on $\frac{dE_{PenPan}}{dt}$ can be approximated as the sum of Equations 18, 21, and 23, ignoring the effect of $\frac{dT_{a}}{dt}$ on $\frac{dR_{n}}{dt}$ and assuming that both *Δ* and *e*_*s*_ are functions solely of *T*_*a*_.

#### Streamflow sensitivity

In the Budyko framework, the annual average partitioning of precipitation between evaporation (*E*) and streamflow (*Q*) is treated as a functional balance between the supply of water from the atmosphere (precipitation; *P*) and the demand for water by the atmosphere (*E*_*o*_). We used Choudhury’s equation^78^ of the Budyko framework which is give as

(Equation 25) $$\begin{array}{c} E=\frac{PE_{o}}{{\left(P^{n}+E_{o}^{n}\right)}^{1/n}} \end{array}$$

where the catchment properties parameter, *n*, represents the net effect on *E* and *Q* of any processes not already encapsulated in *P* and *E*_*o*_, including errors in the representation of *P* and *E*_*o*_. There is currently no definitive process-based understanding of what determines *n* despite several specific investigations.^79^^,^^80^^,^^81^^,^^82^^,^^83^ The recommended default value for *n* was 1.8. To get exact agreement with observations, we optimized the parameter *n* for each sub-catchment by minimizing an unconstrained multivariable function using a derivative-free method.

With the respective partial differentials given by

(Equation 26) $$\begin{array}{c} \frac{\partial E}{\partial P}=\frac{E}{P}\left(\frac{E_{o}^{n}}{P^{n}+E_{o}^{n}}\right) \end{array}$$

(Equation 27) $$\begin{array}{c} \frac{\partial E}{\partial E_{o}}=\frac{E}{E_{o}}\left(\frac{P^{n}}{P^{n}+E_{o}^{n}}\right) \end{array}$$

(Equation 28) $$\begin{array}{c} \frac{\partial E}{\partial n}=\frac{E}{n}\left(\frac{\ln\left(P^{n}+E_{o}^{n}\right)}{n}-\frac{P^{n}\;\ln\;P+E_{o}^{n}\;\ln\;E_{o}}{P^{n}+E_{o}^{n}}\right) \end{array}$$

We used Equation 25 to calculate the changes in *Q* due to changes in climate (*P*, *E*_*o*_) and catchment properties (n). At assumed steady state (i.e., water storage changes being assumed to be negligible), the change in *Q* can be expressed as^77^:

(Equation 29) $$\begin{array}{c} dQ=\left(1-\frac{\partial E}{\partial P}\right)dP-\frac{\partial E}{\partial E_{o}}dE_{o}-\frac{\partial E}{\partial n}dn \end{array}$$

and the relative change in *Q* is as follows:

(Equation 30) $$\begin{array}{c} \frac{dQ}{Q}=\left[\frac{P}{Q}\left(1-\frac{\partial E}{\partial P}\right)\right]\frac{dP}{P}-\left[\frac{E_{o}}{Q}\frac{\partial E}{\partial E_{o}}\right]\frac{dE_{o}}{E_{o}}-\left[\frac{n}{Q}\frac{\partial E}{\partial n}\right]\frac{dn}{n} \end{array}$$

The above terms in square brackets are analytical expressions for the sensitivity coefficients (dimensionless).

### Quantification and statistical analysis

The regional-averaged ensemble means of annual *E*_*PenPan*_ derived from both site observations and gridded datasets are represented as mean ± standard error. The relationship between the observed and modeled *E*_*PenPan*_ at the 79 weather stations was evaluated using an ordinary least squares (OLS) linear regression model. Additionally, the OLS model was employed to examine the relationship between observed and estimated annual streamflow from GloFAS-ERA5.
