# Increasing atmospheric evaporative demand across the Tibetan plateau and implications for surface water resources

**DOI:** 10.1016/j.isci.2025.112598

**Published:** 2025-05-08

**Authors:** Shiqin Xu, Dennis P. Lettenmaier, Tim R. McVicar, Pierre Gentine, Hylke E. Beck, Joshua B. Fisher, Zhongbo Yu, Ningpeng Dong, Akash Koppa, Matthew F. McCabe

## Main text

(iScience *28*, 111623; February 21, 2025)

In the originally published version of this article, the authors mistakenly plotted the ensemble mean, which did not fully capture the E_*PenPan*_ anomalies, in Figure 1B. The authors have now replaced the panel with the correct one in Figure 1B, as well as in the graphical abstract. This adjustment does not affect the findings in the other panels of Figure 1, nor in Figures 2 and 3. The authors regret this error and apologize for any confusion that it may have caused.

**Figure:**
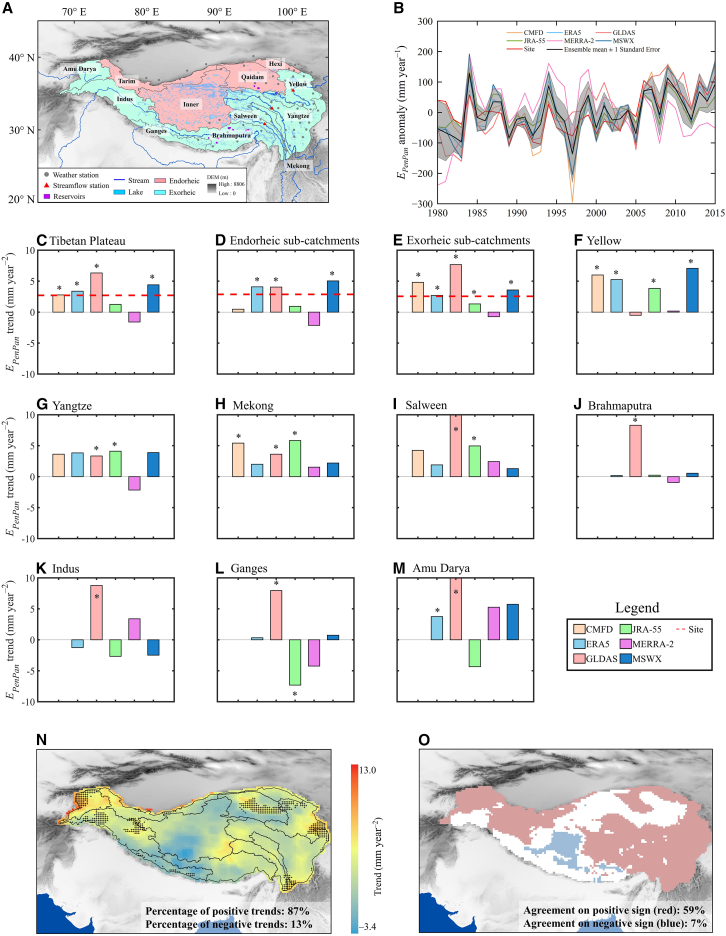
Figure 1. Trends in annual E_*PenPan*_ across the TP for 1980–2015 (corrected)

**Figure:**
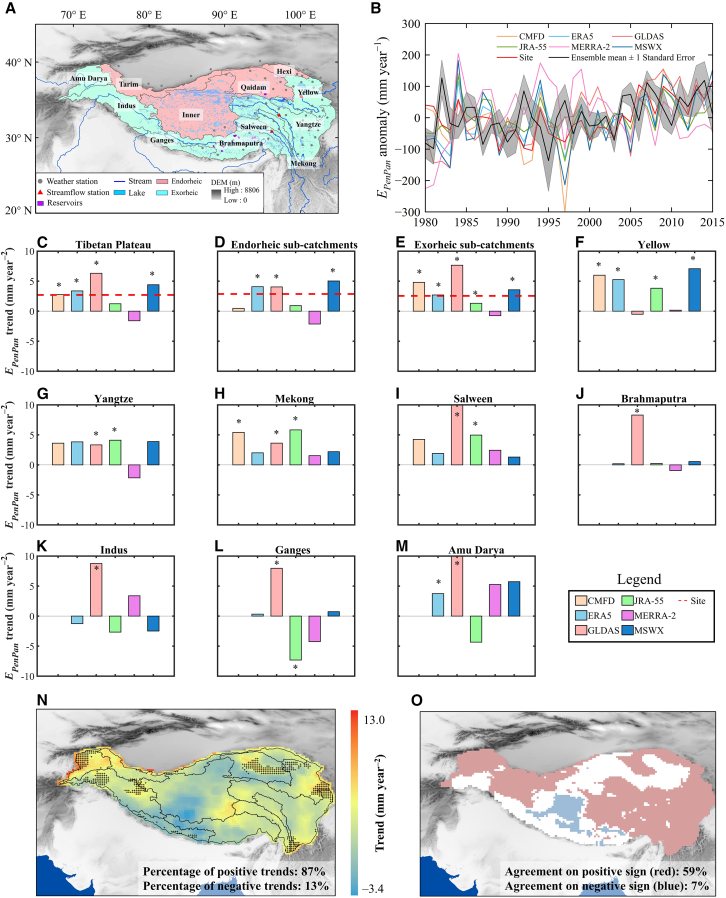
Figure 1. Trends in annual E_*PenPan*_ across the TP for 1980–2015 (original)

