# Supplementary material for: Increasing atmospheric evaporative demand across the Tibetan plateau and implications for surface water resources
Source: iScience. 2024 Dec 18;28(2):111623. doi: 10.1016/j.isci.2024.111623 (PMC11803223; doi:10.1016/j.isci.2024.111623)
Supplement: Document S1. Figures S1–S4 and Tables S1–S9 [file mmc1.pdf]

iScience, Volume *28*

Supplemental information

Increasing atmospheric evaporative demand across the Tibetan plateau and implications for surface water resources

Shiqin Xu, Dennis P. Lettenmaier, Tim R. McVicar, Pierre Gentine, Hylke E. Beck, Joshua B. Fisher, Zhongbo Yu, Ningpeng Dong, Akash Koppa, and Matthew F. McCabe

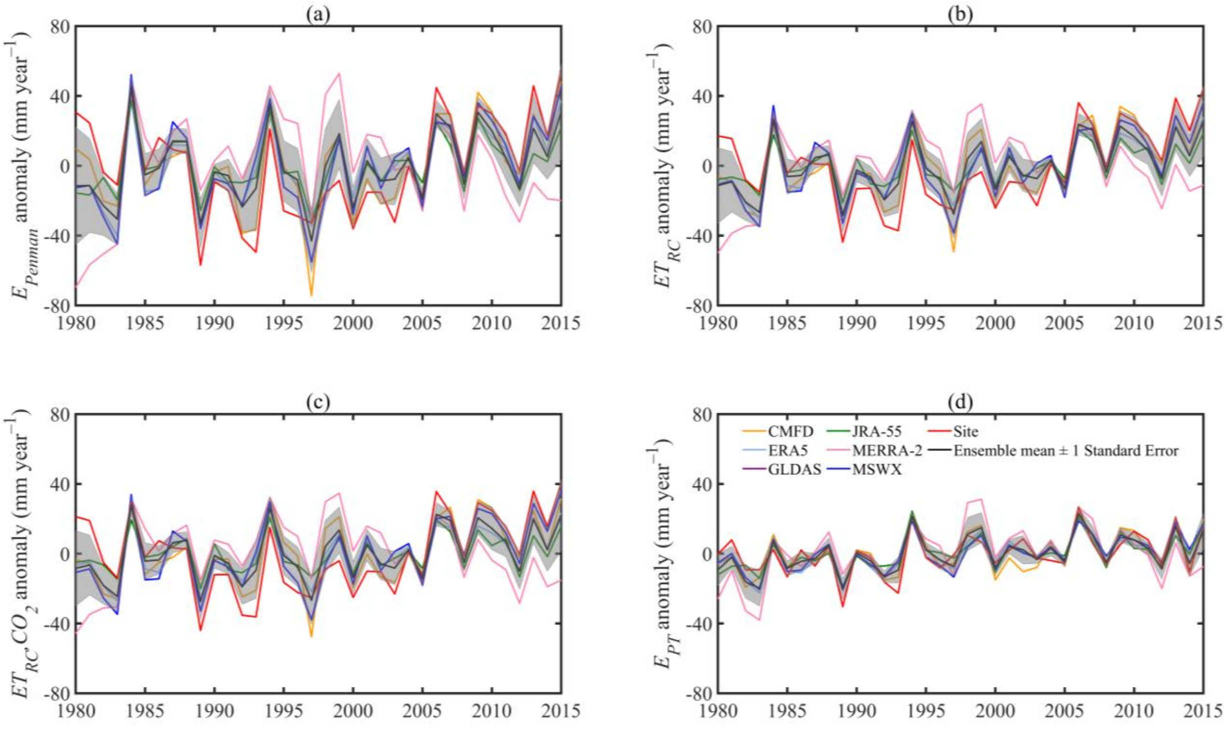

Figure S1 Trends in annual E_o across the TP for 1980–2015. (a) Penman, (b) FAO-56 reference crop, (c) CO_2 adjusted FAO-56 reference crop, and (d) Priestley-Taylor models. The legend on (d) applies to all parts. The regional-averaged ensemble means of annual E_o derived from both site observations and gridded datasets are represented as mean ± standard error.

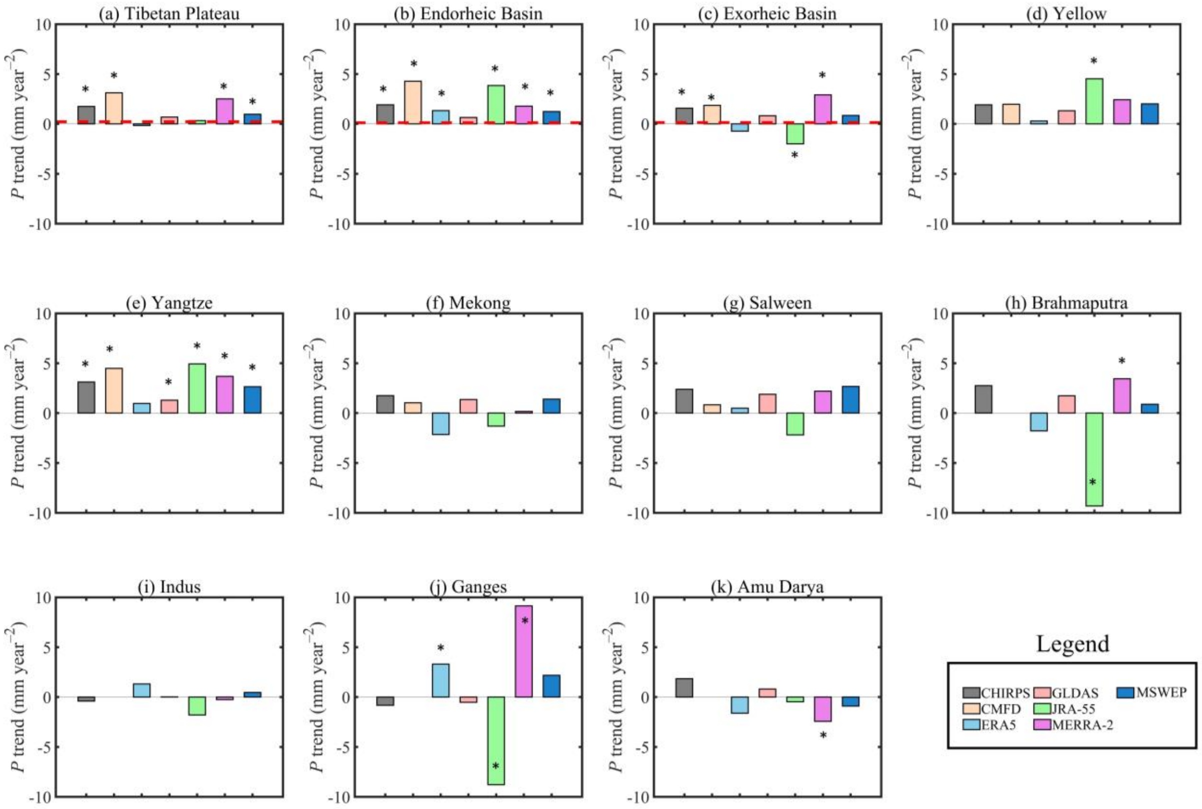

Figure S2 Trends in annual precipitation (*P*) across the entire Tibetan Plateau and sub-catchments over 1980–2015. The code '*' indicates that the *P* trend was significant (p-value < 0.05). The site observations showed insignificant increasing trends (p-value > 0.05) across the entire TP, and endorheic and exorheic catchments. The legend to the right of (k) applies to all parts.

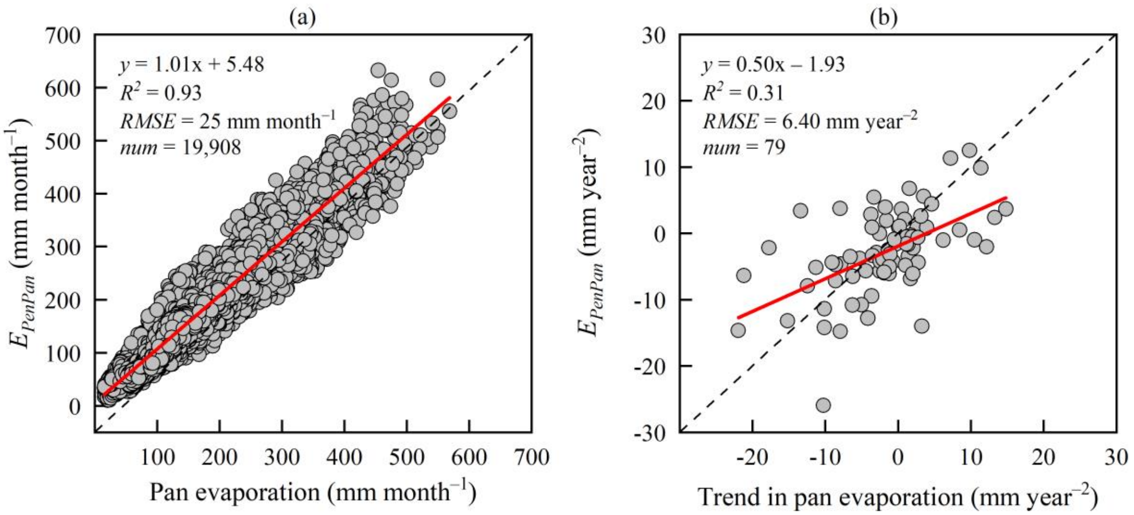

Figure S3 Comparison of the observed and modeled pan evaporation rates (E_{PenPan}) using PenPan model at the 79 weather stations across the Tibetan Plateau. (a) shows the monthly average rate; (b) shows the annual trends. The black dash line is the 1:1 line; the red line is the line of best fit (as given by the equation on each figure part), R^2 is the coefficient of determination; *RMSE* is the Root Mean Squared Error; and *num* is the number of observations.

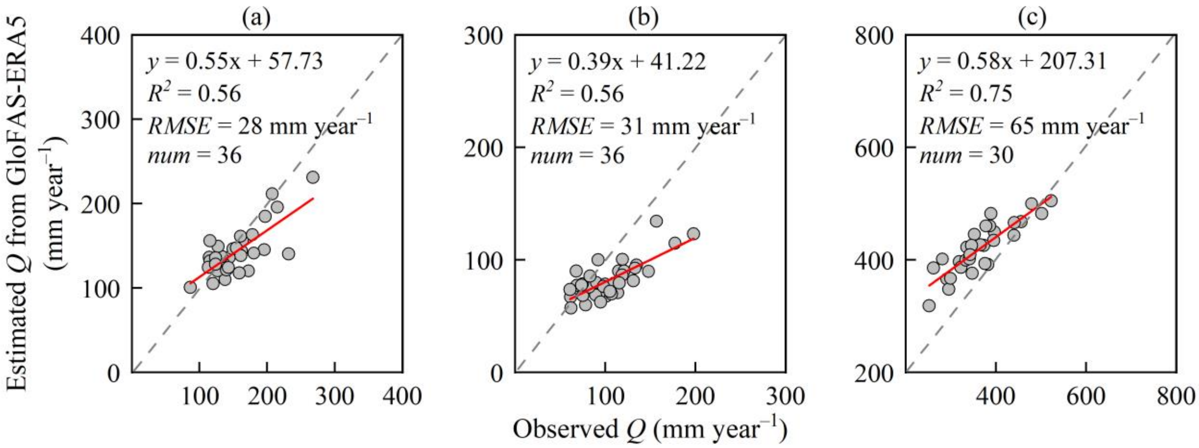

Figure S4 Comparison of the observed and estimated annual streamflow (*Q*) from GloFAS-ERA5. (a) Yellow, (b) Yangtze, and (c) Salween River sub-catchments, respectively. The black dash line is the 1:1 line; the red line is the line of best fit (as given by the equation on each figure part), R^2 is the coefficient of determination; *RMSE* is the Root Mean Squared Error; and *num* is the number of observations.

Table S1 Summary of relevant studies exploring E_o trends and underlying causes in the entire Tibetan Plateau or sub-catchments. Studies are ordered chronologically (according to the year of publication) and then alphabetically within each year. Information of study area and period (Column 2), data source (Column 3), and key results (Column 4) of each study was provided.

Reference	Study area and period	Data source	Key results
[23]	The TP; 1966–2001	Data from 75 weather stations	• The annual ET_{RC} and pan evaporation significantly decreased (p-value < 0.05) while actual annual evapotranspiration averaged from all the catchments increased (p-value < 0.10).<br>• Decreasing trend in ET_{RC} was due to a decrease in wind speed and a decrease in net total radiation, and the increase in air temperature, however, showed little correlation with the declining trends in ET_{RC} and pan evaporation.
[24]	The TP; 1971–2004	Data from 75 weather stations	• Annual ET_{RC} decreased for most part of the TP during the study period.<br>• Wind speed predominated the changes of ET_{RC}.
[25]	The TP; 1970–2005	Data from 75 weather stations	• ET_{RC} decreased by –3.06 mm year^{-2}.<br>• A decrease in wind speed was the dominant factor for the decreasing ET_{RC}.
[26]	The TP; 1970–2009	Data from 75 weather stations	• Annual ET_{RC} decreased on average by –0.69 mm year^{-2} 1970–2009.<br>• The changes in u_2 were found to produce the largest decrease (–0.7 mm year^{-2}) in ET_{RC}, followed by e_a (–0.4 mm year^{-2}) and R_n (–0.1 mm year^{-2}).
[27]	The TP; 1966–2000	Based on Zhang's et al. (2007) estimates.	• Pan evaporation decreased by –4.57 mm year^{-2}.
[28]	The TP; 1961–2010	Data from 81 weather stations	• Regional averaged ET_{RC} series presented a 'zigzag' temporal trend, that is an increasing–decreasing–increasing temporal pattern with two break points in 1973 and 1993.
[29]	The TP; 1981–2010	Data from 80 weather stations	• Regional-averaged ET_{RC} showed decreasing trends.
[15]	The three-river source region of the TP; 1980–2012	Data from 33 weather stations	• Annual ET_{RC} significantly decreased (p-value < 0.05) at a rate of –0.91 mm year^{-2} from 1980 to 2012.<br>• Decrease in R_n was the major reason for the decrease in ET_{RC}.

30	The headwaters of the Yellow River Catchment; 1960–2014	Data from 14 weather stations	• Average annual ET_{RC} showed significant decreasing trend (p-value < 0.05) by –0.32 mm year^{-2}.<br>• Reduction of wind speed was the major driving force for decreasing trends in annual and seasonal ET_{RC}.
31	The TP; 1970–2011	Data from 77 weather stations	• E_{PenPan} showed significant decreasing trends (p-value < 0.05) in 1970–2001 and insignificant increasing trends in 2002–2011.<br>• Decreasing R_n and wind speed contributed to the decreasing of E_{PenPan} in 1970–2001, and the increasing vapour pressure deficit contributed to the increasing of E_{PenPan} in 2002–2011.

Table S2 Observed E_{PenPan} long-term climatology in 1980–2015 (mm year^{-1}) and its annual trend (mm year^{-2}) over the same period for the entire Tibetan Plateau and sub-catchments. The code '*' indicates that time series of annual E_{PenPan} showed significant trend (p-value < 0.05). N/A denotes 'Not Applicable' as the China Meteorological Forcing Dataset (CMFD) does not cover those sub-catchments. Given the limited and uneven distribution of the China Meteorological Administration sites, we opted not to include site-specific observations in the analysis at the exorheic sub-catchments.

Catchment	CMFD		ERA5		GLDAS		JRA55		MERRA2		MSWX		Site	
	Climatology	Trend	Climatology	Trend	Climatology	Trend	Climatology	Trend	Climatology	Trend	Climatology	Trend	Climatology	Trend
Tibetan Plateau	2,193	+2.8*	1,907	+3.4*	2,186	+6.3*	1,933	+1.2	2,342	–1.6	2,231	+4.4*	1,937	+2.7*
Endorheic catchments	2,369	+0.5	2,274	+4.1*	2,475	+4.0*	2,066	+0.9	2,578	–2.1	2,542	+5.0*	2,380	+2.9*
Exorheic catchments	2,032	+4.8*	1,649	+2.7*	1,980	+7.7*	1,832	+1.3*	2,173	–0.7	2,013	+3.6*	2,013	+3.6*
Yellow	1,766	+6.0*	1,743	+5.3*	1,819	–0.5	1,641	+3.8*	1,996	+0.2	1,961	+7.1*	N/A	N/A
Yangtze	1,899	+3.6	1,791	+3.8	1,784	+3.3*	1,737	+4.1*	2,110	–2.2	2,014	+3.9	N/A	N/A
Mekong	1,879	+5.4*	1,652	+2.0	1,594	+3.6*	1,904	+5.8*	2,171	+1.5	2,079	+2.2	N/A	N/A
Salween	1,878	+4.2	1,553	+1.9	1,728	+9.9*	1,828	+5.0*	2,160	+2.4	1,843	+1.3	N/A	N/A
Brahmaputra	N/A	N/A	1,712	+0.2	1,906	+8.3*	1,947	+0.2	2,218	–0.9	2,040	+0.5	N/A	N/A
Indus	N/A	N/A	2,233	–1.3	1,907	+8.8*	2,015	–2.7	2,679	+3.4	2,822	–2.5	N/A	N/A
Ganges	N/A	N/A	1,631	+0.3	2,041	+8.0*	1,848	–7.3*	2,396	–4.2	2,090	+0.7	N/A	N/A
Amu Darya	N/A	N/A	1,551	+3.7*	2,550	+23.7*	1,924	–4.3	2,196	+5.3	1,977	+5.7*	N/A	N/A

Table S3 Observed E_{Penman} long-term climatology (mm year^{-1}) in 1980–2015 and its annual trend (mm year^{-2}) over the same period for the entire Tibetan Plateau and sub-catchments. The code '*' indicates that time series of annual E_{Penman} showed significant trend (p-value < 0.05). N/A denotes 'Not Applicable' as the China Meteorological Forcing Dataset (CMFD) does not cover those sub-catchments. Given the limited and uneven distribution of the China Meteorological Administration sites, we opted not to include site-specific observations in the analysis at the exorheic sub-catchments.

Catchment	CMFD		ERA5		GLDAS		JRA55		MERRA2		MSWX		Site	
	Climatology	Trend	Climatology	Trend	Climatology	Trend	Climatology	Trend	Climatology	Trend	Climatology	Trend	Climatology	Trend
Tibetan Plateau	1,272	+0.8	1,235	+0.9*	1,259	+2.2*	1,311	+0.5	1,400	–0.1	1,237	+1.1*	1,455	+0.6*
Endorheic catchments	1,300	+0.3	1,314	+1.2*	1,301	+1.6*	1,312	+0.2	1,420	–0.4	1,309	+1.3*	1,612	+0.4*
Exorheic catchments	1,246	+1.3*	1,180	+0.8*	1,229	+2.6*	1,308	+0.6*	1,385	–0.1	1,187	+0.9*	1,187	+0.9*
Yellow	1,144	+1.8*	1,168	+1.3*	1,132	–0.1	1,229	+1.0*	1,297	+0.1	1,133	+1.5*	N/A	N/A
Yangtze	1,207	+0.9	1,211	+1.2	1,126	+1.7*	1,258	+0.9*	1,335	–0.2	1,170	+1.1	N/A	N/A
Mekong	1,214	+1.3*	1,203	+0.6	1,131	+1.8*	1,359	+1.9*	1,398	+0.9	1,195	+0.6	N/A	N/A
Salween	1,229	+1.5	1,189	+0.4	1,173	+3.9*	1,328	+1.5*	1,393	+1.0	1,146	+0.4	N/A	N/A
Brahmaputra	N/A	N/A	1,241	0	1,283	+2.6*	1,376	+0.5	1,460	+0.1	1,243	+0.1	N/A	N/A
Indus	N/A	N/A	1,398	–0.1	1,302	+3.9*	1,383	–0.6	1,530	+0.6	1,488	–0.4	N/A	N/A
Ganges	N/A	N/A	1,230	0	1,317	+2.8*	1,357	–1.5*	1,531	–0.9	1,294	+0.2	N/A	N/A
Amu Darya	N/A	N/A	1,100	+1.2*	1,287	+6.1*	1,249	–1.0	1,282	+1.3	1,135	+1.5*	N/A	N/A

Table S4 Observed ET_{RC} long-term climatology in 1980–2015 (mm year^{-1}) and its annual trend (mm year^{-2}) over the same period for the entire Tibetan Plateau and sub-catchments. The code '*' indicates that time series of annual ET_{RC} showed significant trend (p-value < 0.05). N/A denotes 'Not Applicable' as the China Meteorological Forcing Dataset (CMFD) does not cover those sub-catchments. Given the limited and uneven distribution of the China Meteorological Administration sites, we opted not to include site-specific observations in the analysis at the exorheic sub-catchments.

Catchment	CMFD		ERA5		GLDAS		JRA55		MERRA2		MSWX		Site	
	Climatology	Trend	Climatology	Trend	Climatology	Trend	Climatology	Trend	Climatology	Trend	Climatology	Trend	Climatology	Trend
Tibetan Plateau	840	+1.0*	819	+0.8*	769	+1.7*	875	+0.5*	935	+0.1	792	+1.1*	1002.1	+0.8*
Endorheic catchments	852	+0.6	860	+1.0*	805	+1.2*	857	+0.3	940	–0.1	839	+1.1*	1123.9	+0.6
Exorheic catchments	829	+1.4*	790	+0.7*	742	+2.1*	884	+0.5*	930	+0.2	758	+0.8*	758.3	+0.8*
Yellow	747	+2.0*	757	+1.1*	667	+0.1	851	+0.7*	880	+0.3	706	+1.6*	N/A	N/A
Yangtze	768	+1.2*	766	+1.0*	664	+1.0*	847	+0.9*	885	0	718	+1.0	N/A	N/A
Mekong	827	+1.0	805	+0.5*	644	+1.2*	943	+1.4*	949	+0.7	755	+0.7	N/A	N/A
Salween	820	+1.3*	783	+0.3*	669	+2.8*	907	+1.1*	925	+0.9	700	+0.3	N/A	N/A
Brahmaputra	N/A	N/A	836	+0.1*	757	+2.6*	930	+0.4	972	+0.2	798	+0.2	N/A	N/A
Indus	N/A	N/A	912	0	724	+2.9*	858	–0.3	970	+0.6	931	–0.2	N/A	N/A
Ganges	N/A	N/A	833	+0.2	784	+2.6*	914	–0.9*	1,011	–0.5	831	+0.3	N/A	N/A
Amu Darya	N/A	N/A	747	+0.8*	848	+4.8*	815.3	–1.4*	859.2	+1.1	738	+1.1*	N/A	N/A

Table S5 Observed E_{PT} long-term climatology in 1980–2015 (mm year^{-1}) and its annual trend (mm year^{-2}) over the same period for the entire Tibetan Plateau and sub-catchments. The code '*' indicates that time series of annual E_{PT} showed significant trend (p-value < 0.05). N/A denotes 'Not Applicable' as the China Meteorological Forcing Dataset (CMFD) does not cover those sub-catchments. Given the limited and uneven distribution of the China Meteorological Administration sites, we opted not to include site-specific observations in the analysis at the exorheic sub-catchments.

Catchment	CMFD		ERA5		GLDAS		JRA55		MERRA2		MSWX		Site	
	Climatology	Trend	Climatology	Trend	Climatology	Trend	Climatology	Trend	Climatology	Trend	Climatology	Trend	Climatology	Trend
Tibetan Plateau	974	+0.5*	1,005	+0.5*	975	+1.3*	1,100	+0.4*	1,111	+0.4	929	+0.5*	1,057	+0.4
Endorheic catchments	961	+0.2	1,000	+0.6*	933	+1.2*	1,055	+0.3	1,066	+0.4	921	+0.5*	1,046	+0.5*
Exorheic catchments	987	+0.6*	1,008	+0.4*	1,003	+1.3*	1,127	+0.5*	1,141	+0.4	933	+0.5*	933	+0.5*
Yellow	919	+1.0*	962	+0.6*	916	+0.4	1,069	+0.4	1,060	+0.7	855	+0.7*	N/A	N/A
Yangtze	950	+0.7	984	+0.7*	885	+1.0*	1,064	+0.3	1,068	+0.6	874	+0.6*	N/A	N/A
Mekong	990	+0.3	1,047	+0.5	971	+1.2*	1,174	+1.0*	1,159	+0.8	935	+0.3	N/A	N/A
Salween	1,000	+1.0*	1,043	+0.2	987	+1.9*	1,142	+0.9*	1,143	+1.0*	923	+0.1	N/A	N/A
Brahmaputra	N/A	N/A	1,069	0	1,090	+1.1*	1,185	+0.7*	1,225	+0.5	999	0	N/A	N/A
Indus	N/A	N/A	1,110	+0.1	1,098	+2.8*	1,151	0	1,167	+0.3	1,068	+0.1	N/A	N/A
Ganges	N/A	N/A	1,070	+0.1	1,075	+1.3*	1,186	0	1,272	+0.1	1,049	+0.1	N/A	N/A
Amu Darya	N/A	N/A	942	+0.8*	940	+1.5*	1,040	–0.1	1,014	+0.4	893	+0.8*	N/A	N/A

Table S6 Observed $ET_{RC,CO2}$ long-term climatology in 1980–2015 (mm year^{-1}) and its annual trend (mm year^{-2}) over the same period for the entire Tibetan Plateau and sub-catchments. The code '*' indicates that time series of annual $ET_{RC,CO2}$ showed significant trend (p-value < 0.05). N/A denotes 'Not Applicable' as the China Meteorological Forcing Dataset (CMFD) does not cover those sub-catchments. Given the limited and uneven distribution of the China Meteorological Administration sites, we opted not to include site-specific observations in the analysis at the exorheic sub-catchments.

Catchment	CMFD		ERA5		GLDAS		JRA55		MERRA2		MSWX		Site	
	Climatology	Trend	Climatology	Trend	Climatology	Trend	Climatology	Trend	Climatology	Trend	Climatology	Trend	Climatology	Trend
Tibetan Plateau	831	+0.8*	811	+0.6*	757	+1.4*	868	+0.3	926	–0.1	785	+1.1*	1,000	+0.6
Endorheic catchments	842	+0.4	850	+0.7*	791	+0.8*	849	+0.1	930	–0.4	831	+1.1*	1,126	+0.4
Exorheic catchments	821	+1.2*	784	+0.5*	731	+1.8*	879	+0.3	923	–0.1	752	+0.8*	752	+0.8*
Yellow	739	+1.8*	748	+0.9*	656	–0.2	848	+0.7*	874	+0.1	699	+1.6*	N/A	N/A
Yangtze	759	+0.9*	757	+0.7	653	+0.7	841	+0.7*	877	–0.3	711	+1.0	N/A	N/A
Mekong	822	+0.8	799	+0.4	633	+0.9*	939	+1.3*	942	+0.5	749	+0.7	N/A	N/A
Salween	813	+1.1*	776	+0.1	658	+2.5*	902	+1.0*	916	+0.6	693	+0.3	N/A	N/A
Brahmaputra	N/A	N/A	829	–0.1	745	+2.3*	923	+0.2	963	0.0	792	+0.2	N/A	N/A
Indus	N/A	N/A	903	–0.3	711	+2.6*	848	–0.7	956	+0.3	922	–0.2	N/A	N/A
Ganges	N/A	N/A	827	0.0	771	+2.2*	907	–1.0*	1,001	–0.8	825	+0.3	N/A	N/A
Amu Darya	N/A	N/A	742	+0.7*	837	+4.5*	808	–1.6*	852	+0.9	732	+1.1*	N/A	N/A

Table S7 Sensitivity of relative changes in streamflow (*Q*) to the relative change in precipitation (*P*), atmospheric evaporative demand (E_o), and catchment properties (*n*) (dimensionless) based on the water budget integrated regionally over the seven sub-catchments for which *Q* were available.

Number	E_o model	Sensitivity of *Q* to *P*	Sensitivity of *Q* to E_o	Sensitivity of *Q* to *n*
1	Penman model	1.7	–0.7	–1.6
2	FAO-56 reference crop evapotranspiration model	1.9	–0.9	–1.7
3	CO_2 adjusted FAO-56 reference crop evapotranspiration model	1.9	–0.9	–1.7
4	Priestley-Taylor model	1.8	–0.8	–1.7
5	PenPan model	1.5	–0.5	–1.4
6	Radiation-driven model	1.6	–0.6	–1.5

Table S8 Details of the exorheic sub-catchments in the Tibetan Plateau. N/A denotes 'Not Applicable' as streamflow observations weren't readily available for these catchments from the Ministry of Water Resources of China. Data source of the glacier areas were provided.

	Yellow	Yangtze	Mekong	Salween	Brahmaputra	Ganges	Indus	Amu Dayra
Control station	Tangnaihai	Zhimenda	N/A	Jiayuqiao	N/A	N/A	N/A	N/A
Catchment area (km^2)	123,023	140,073	90,441	75,506	347,506	85,273	319,123	125,633
Glacier area (km^2)	129	1,249	219	1,041	4,261	5,885	27,073	9,561
Temporal coverage of streamflow	1980–2015	1980–2015	1980–2015	1981–2010	1980–2015	N/A	1980–2015	1980–2015

Table S9 Summary of the data used herein. Datasets are ordered alphabetically. N/A denotes 'Not Applicable'.

Datasets	Temporal extent	Temporal resolution	Spatial resolution
CHIRPS-V2.0	1980–present	Daily	0.05
CMFD	1979–2018	Monthly	0.1°
GLDAS NOAH025 M	1980–2014	Monthly	0.25°
GloFAS-ERA5	1979–present	Daily	0.05°
GTOPO30 DEM	N/A	N/A	0.1°
ERA5	1980–present	Monthly	0.1°
MERRA-2	1980–present	Monthly	0.50° × 0.625°
JRA-55	1958–present	Monthly	0.5625° × 0.5625°
MSWEP-V2	1979–present	Monthly	0.1°
MSWX	1979–present	Monthly	0.1°
Weather station observation	1980–2015	Daily	Point, representative of a larger often unknown area
